# Dispersal ability and niche breadth influence interspecific variation in spider abundance and occupancy

**DOI:** 10.1098/rsos.230051

**Published:** 2023-05-10

**Authors:** Daniel Suárez, Paula Arribas, Nuria Macías-Hernández, Brent C. Emerson

**Affiliations:** ^1^ Island Ecology and Evolution Research Group, Institute of Natural Products and Agrobiology (IPNA-CSIC), C/Astrofísico Francisco Sánchez 3, La Laguna, Tenerife, Canary Islands 38206, Spain; ^2^ School of Doctoral and Postgraduate Studies, University of La Laguna, 38200 La Laguna, Tenerife, Canary Islands 38200, Spain; ^3^ Departamento de Biología Animal, Edafología y Geología, Universidad of La Laguna, 38200 La Laguna, Tenerife, Canary Islands 38200, Spain; ^4^ Laboratory for Integrative Biodiversity Research (LIBRe), Finnish Museum of Natural History LUOMUS, University of Helsinki, 00014 Helsinki, Finland

**Keywords:** dispersal, niche, abundance, occupancy, arthropod, spider

## Abstract

The relationship between species local abundance and their regional distribution (occupancy) is one of the most extensively recognized and investigated patterns in ecology. While exceptions exist, the generally held model is that locally abundant species also tend to be more widespread geographically. However, there is only a limited understanding of both the mechanisms driving this relationship, and their scale dependency. Here we use occupancy and abundance data for 123 species of spider from across the Canary Islands to understand how both dispersal ability and niche breadth might mediate variation among species for local abundance and occupancy. We test the predictions that (i) dispersal ability explains variation among species for both abundance and occupancy, and (ii) species with a higher degree of habitat specialization, reflecting more limited niche breadth, will have both higher occupancy and abundance. We find no evidence within habitat patches for an effect of dispersal ability on either local abundance or site occupancy, while across all patches species with higher dispersal ability tend to occupy more sites. Species largely restricted to laurel forests have higher abundance than species with broader niche breadth, but similar occupancy. The study revealed that dispersal ability and niche breadth were significant predictors of the abundance–occupancy relationship, highlighting the importance of both factors for understanding patterns of abundance and occupancy among spider species.

## 1. Introduction

Since its original theoretical development based on multi-taxon evidence [1], the relationship between species' local population densities (abundance) and their regional distribution (occupancy), i.e. the ‘abundance–occupancy relationship’ (AOR), has been one of the most extensively recognized and investigated patterns in ecology [2–4]. Both variables are positively related, with widespread species typically being more locally abundant, while species with more limited distributions are typically found at lower abundance. Occupancy can be measured as the distance/area between the outermost limits to a species occurrence (i.e. the extent of occurrence), or the area over which a species is found (i.e. area of occurrence) [5]. The latter will tend to be smaller, as species do not occupy all areas within the geographical limits to their occurrence [6]. The distinction between those measures is important as it has been shown that the extent of occurrence usually shows no correlation with abundance [2]. Regarding abundance, in local scale analyses it can be measured directly using site population counts. However, local abundance can also be estimated by dividing the total population size of a given domain by the number of occupied sites within that domain. The latter measure may give rise to a number of issues summarized by Borregaard & Rahbek [7]. Attempts have been made to disentangle the factors that may explain the AOR relationship, with Gaston *et al*. [3] suggesting up to eight mechanisms may drive this positive relationship, with none of them being necessarily mutually exclusive or even independent. The first two mechanisms are due to statistical artefacts, i.e. sampling bias (insufficient sampling effort leads to a systematic underestimation of the range sizes of species with lower local densities) and phylogenetic non-independence (due to phylogenetic relatedness, species do not constitute independent data points, thus inflating degree of freedom for testing statistical significance) [3]. The remaining six are essentially biological and can be grouped in three categories: range position, resource and population dynamic explanations [4]. All six mechanisms have been evaluated and have gained some support, thus leading to a model where a positive AOR may result from the action of several mechanisms, with varying importance depending upon the system [8]. However, particular emphasis has been placed on the potential role of species dispersal ability and niche breadth as factors shaping AORs.

From a neutral perspective, species that have a high dispersal ability are more prone to be widespread ([6] and references therein) as individuals are more able to colonize favourable habitat patches for resource exploitation. Although there are theoretical predictions that suggest that dispersal ability should be an important predictor of relative abundance (e.g. [7–13]), Levine & Murell [14] have suggested that even though dispersal ability influences the rate of arrival, other factors such as competition or geographical habitat variation may be more important in controlling relative abundance patterns. Thus, differences in dispersal ability have been a central focus in recent AOR studies, and Freckleton *et al*. [15] have developed a model incorporating colonization and dispersal within patchy landscapes to explore AORs. Within this model, both the strength and the shape of the AOR are affected by the ability of species to disperse between habitat patches. For taxa with low-dispersal ability, AORs are predicted to be absent, while for species with moderate dispersal ability, regional populations are predicted to exhibit classic metapopulation dynamics. Empirical tests of this model using marine macroinvertebrates have demonstrated that, for a given population size, more dispersive taxa exhibit greater regional occupancy, resulting in less aggregated distributions at a local scale [16,17].

With regard to the role of niche as an explanatory variable for the AOR, two hypotheses have been proposed. The ‘niche breadth’ hypothesis [1] posits that species with broader environmental tolerances (generalists) are able to use a wider variety of resources and will thus be locally more abundant and spatially widespread. However, the ‘niche position’ hypothesis [18], which has received much empirical support (e.g. [19,20,21]), asserts that species that are able to efficiently exploit the most common environmental conditions within a given habitat (i.e. specialized to a dominant habitat) will be more locally abundant and widespread than generalist species. The niche position hypothesis can be seen as analogous to the ‘jack-of-all-trades is master of none’ [22] within the context of a particular habitat.

Although factors shaping abundance and occurrence within a given assemblage have been broadly addressed, their interplay with spatial scale has been given considerably less attention. He & Gaston [23] first studied how the AOR curve changes at different spatial grains using a dataset of tree species in a tropical rainforest, showing that curve-fitting is more linear when the spatial grain is lower. More recently, Steenweg *et al*. [24] revealed that curve-fitting is not only affected by spatial grain (size of the grid cells that are used to discretize continuous space), but also the sampling unit (areal versus point sampling). If occupancy is measured at broader sampling units, then the AOR is unaffected by spatial grain, while at narrower sampling units, the AOR follows the pattern described by He & Gaston [23]. Empirically, Gaston *et al*. [25] tested the fit of the AOR of soil and canopy arthropod assemblages at three different scales within an oceanic archipelago framework: a small reserve, the island of Terceira, and seven islands in the Azorean archipelago. A positive AOR was found at all three scales, but with no further explanation for the potential drivers of this pattern, and to date there is still limited data to explore the drivers of abundance and occupancy at different scales.

Through the implementation of a standardized field sampling and molecular identification protocol to characterize laurel forest spider assemblages within the archipelago setting of the Canary Islands, we explore the influence of both dispersal ability and niche breadth on local abundance and occupancy patterns at two different spatial scales, within and among habitat patches. Our sampling framework comprises laurel forest habitat patches (islands) that have never been connected, thus providing a natural dispersal filter among patches. We sample across 31 laurel forest sites within four habitat patches to test the hypothesis that dispersal ability explains variation in abundance and occupancy among species, wherein good dispersers are both more abundant and more widespread. We also evaluate the influence of niche breadth on both abundance and occupancy. More specifically, we hypothesize that species more specialized to laurel forest conditions will be both more abundant and more widespread across habitat patches. Both hypotheses are tested at the island and archipelago scales.

## 2. Material and methods

### 2.1. Field sampling

**Figure 1.**
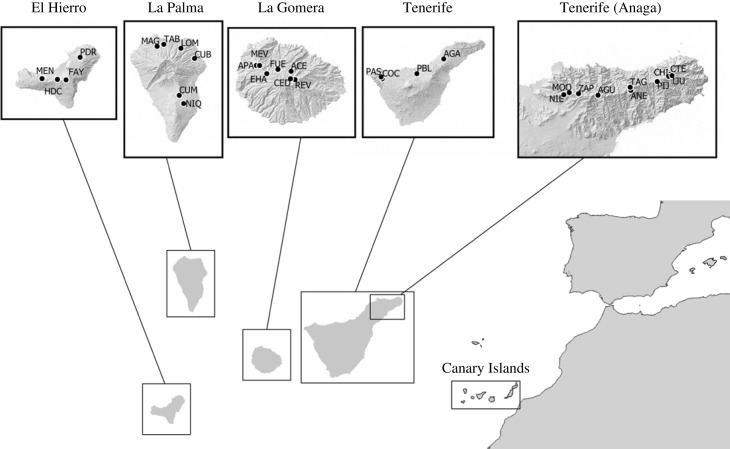
Sampling sites within the laurel forests of the Canary Islands. Sampling sites are labelled with three-letter codes (see electronic supplementary material, table S1 for further details).

A total of 31 sites measuring 50 × 50 m were sampled within laurel forest habitat across the four western islands of the Canary archipelago: Tenerife (14), La Gomera (7), La Palma (6) and El Hierro (4) (figure 1; electronic supplementary material, table S1). Sampling was restricted to these islands as the remaining islands in the archipelago have only limited or no laurel forest extension [26]. Within each site, a standardized field sampling and sample processing protocol was applied [27], encompassing 16 h of active sampling (foliage beating, vegetation sweeping and active searching) together with 48 pitfall traps that were set for 14 days, and extraction from leaf litter using Berlese apparatus. This combination of techniques facilitates the collection of species that are highly mobile as well as those that are either less mobile or typically hidden. Sampling was carried out from 2012 to 2020 between the months of November and May. Samples collected in the field were preserved in absolute ethanol at −20°C until further examination.

### 2.2. Mitochondrial DNA sequencing for species delimitation

Spatially explicit quantification of local arthropod species richness and diversity can be particularly challenging, frequently complicated by the lack of or incomplete regional species lists, limited taxonomic expertise and potential cryptic diversity (e.g. [28,29,30]). In response to this taxonomic impediment, the protocol of Emerson *et al*. [27], which combines parataxonomy with molecular identification, was followed. Spider specimens were classified into parataxonomic units (PUs) by direct examination of external morphology under a binocular lens. As in Salces-Castellano *et al.* [31], we defined two types of PU within each plot: (i) type I are confidently recognized in other plots (e.g. genus is monospecific or with straightforward taxonomy), and (ii) type II are distinct within the focal plot but difficult to cross reference with other plots (e.g. genera with complex or poorly understood taxonomy). PUs were named numerically, with type II PUs denoted with the addition of an ‘x’ to the numerical code. Up to four individuals per PU per site were selected for DNA extraction and sequencing. Depending upon specimen size, a single leg, several legs or prosome were digested using a Chelex protocol [32]. The 5′ region (658 bp) of the mtDNA COI gene was amplified using the primers LCO1490 and HCO2198 [33]. Polymerase chain reaction (PCR) reaction conditions were as follows: initial denaturation at 94°C for 2 min, followed by 40 cycles of 94°C for 30 s, 42–46°C for 35 s and 72°C for 45 s, and a final extension of 72°C for 5 min. One–2 µl of diluted (1/10) DNA extract was amplified with 24–23 µl of PCR mix (for a total volume of 25 µl), comprising 14.4 µl of water, 2.5 µl of 10x NH_4_ buffer (Bioline), 1.5 µl of 50 mM MgCl_2_ (Bioline), 2 µl of 2.5 mM dNTPs (Bioline), 0.5 µl of BSA (20 mg ml^−1^), 1 µl of each primer (10 µM) and 0.1 µl of Taq polymerase (BIOTAQ). PCR products were purified using Exonuclease I and rAPid alkaline phosphatase and then sequenced using the Sanger DNA sequencing service of Macrogen (www.macrogen.com) with either the forward or reverse primer, or both primers in the event of insufficient read length from a single one. Sequences were then edited in Geneious version 2021.1.1 [34]. To identify presumed biological species (PBS) [27], a custom R script ([35], https://github.com/asalcescastellano/Divergence-threshold) was used to produce an unweighted pair group method with arithmetic mean tree from pairwise K2P distances, using an alignment of all sequences from all PUs. This script first implements a conservative maximum intraspecific divergence threshold of 6.8% [36] to create individual alignments (lineages). Under this divergence threshold, it is assumed that all individuals of a given biological species will be represented within a single lineage, but that a given lineage may comprise more than one biological species. The script then generates a table summarizing: (i) the PU composition within each lineage, (ii) for each PU within a lineage, the proportion of individuals from that PU included within the lineage, and (iii) when a lineage comprises individuals from more than one PU, the presence or absence of monophyly among individuals from the same PU. Lineages exclusively comprising all individuals within a given PU were inferred to represent a single PBS. Individuals from PUs that segregated across more than one lineage were revised to evaluate potential PU assignment error or possible laboratory contamination, with the removal of any sequences ascribed to the latter. When two or more PUs segregated within the same lineage, individuals were revised morphologically, and in the absence of any differences (as might be expected among type II PUs—see above) the lineage was inferred to represent a single PBS. For lineages comprising two or more morphologically distinct PUs, and where PUs were phylogenetically structured within the lineage, PUs were inferred to represent different PBS. Morphologically distinct PUs that showed no clear segregation with regard to mtDNA variation were inferred to represent recent speciation with incomplete lineage sorting and/or gene flow, and were removed for subsequent analyses. PBS were then taxonomically assigned to species or genus level based on GenBank and BOLD Systems search results, together with additional information and dichotomous keys from Arnedo *et al*. [37,38] for *Dysdera* Latreille, 1804; Bosmans *et al*. [39] for *Clubiona* Latreille, 1804; Dimitrov & Ribera [40] for *Pholcus* Walckenaer, 1805; Lissner [41] for *Rhomphaea* L. Koch, 1872; Muster *et al*. [42] for *Pulchellodromus* Wunderlich, 2012 and Wunderlich [43–46] for the remaining genera. This process of identification was further supported with reference to the arachnological collection of the Department of Animal Biology, University of La Laguna, Canary Islands, Spain (coll. DZUL) and that of the Island Ecology and Evolution Group (GEEI) of the Instituto de Productos Naturales y Agrobiología (IPNA-CSIC), Tenerife, Canary Islands, Spain (coll. GEEI).

PBS abundances were extrapolated from PU sequencing results to unsequenced PU individuals. Within a given PU, if all sequenced individuals were assigned to a single PBS, then all unsequenced individuals for the PU were assigned to that PBS. If individuals from a given PU were assigned to more than one PBS, then within each site the proportion of each PBS (within the PU) was estimated (from sequenced specimens) and unsequenced individuals were assigned to each PBS within each site accordingly. As a hypothetical example, if 10 individuals sequenced from a given PU within a given site are assigned to different PBS (e.g. three specimens assigned to PBS001 and seven to PBS002), then unsequenced individuals are assigned following the same proportions (e.g. 30% to PBS001 and 70% to PBS002). If the result yields a decimal number, then it is rounded to an integer. PBS (hereafter species) were categorized for dispersal ability, considering the potential for juvenile stages to be passively dispersed by air currents, while suspended from silk threads, henceforth referred to as ‘ballooning’, which has been broadly used as a proxy for dispersal ability [47–49]. Species were categorized as either ballooning or non-ballooning following a family-level classification established by Carvalho & Cardoso [50]. We adopt the concept of ‘niche breadth’ as a proxy for the Grinnellian realized niche, i.e. variability in environmental conditions recorded where a species occurs [51]. A two-step process was used to categorize species as either ‘non-specialist’ or ‘specialist’, based on their affinity to the laurel forest habitat, i.e. whether they are present in a broad range of habitats or restricted mainly to laurel forest. As a first step, the species list was assessed by five Canary Island spider specialists (Miquel Arnedo (University of Barcelona), Jørgen Lissner (Aarhus University), Pedro Oromí (University of La Laguna) and the authors D.S. and N.M.), to categorize species as either laurel forest specialists or non-specialists based on their knowledge of the biology of the species. Only species that were categorized by at least two experts were considered in this step. When all experts consensually categorized a given species, then that category was assigned. In the absence of a consensus, the majority proposed category was assigned. In the absence of a majority opinion, the given species was assigned a category in the subsequent step. In a second step, species association to the laurel forest was quantified using distribution records within the Biodiversity Data Bank of the Canary Islands (https://www.biodiversidadcanarias.es/biota/). The total number of 500 × 500 m cells occupied by a species in the archipelago was quantified and the percentage of those cells corresponding with laurel forest habitat was then calculated. For the subset of species that were categorized by the panel of experts as laurel forest specialists, species were ranked from highest to lowest per cent laurel forest occupancy, and the percentage corresponding to the 50% quantile was used for the categorization of the species non-characterized in the first step (i.e. above this percentage, species were considered as ‘specialists’, whereas below it, species were categorized as ‘non-specialist’). Additionally, 10%, 25% and 75% quantiles were also used for niche breadth categorization to explore the robustness of further inferences. Finally, further analyses were also performed using only the subset of species that were unambiguously categorized in step one (panel of experts).

### 2.3. Abundance and occupancy analyses

For each species, local abundance was calculated as (i) mean site abundance (i.e. the sum of all individuals divided by the number of occupied sites at both island and archipelago scales, with species being the replicate unit), and (ii) individual site abundance (i.e. the sum of all individuals within each site, with site being the replicate unit). Occupancy was calculated both across islands (i.e. the presence or absence of each species within each of the islands) and across sites (i.e. the presence or absence of each species within each site). The latter was again calculated both at the archipelago (across all sites) and at the island (across all sites within a given island) scales. Species richness was compared between dispersive and non-dispersive species, as well as between non-specialist and specialist species, using a generalized linear mixed model (glmer function, lme4 package [52]), implementing island as a nesting factor. Abundance and occupancy were compared between dispersive and non-dispersive species, as well as between non-specialist and specialist species, using both mean site abundance and individual site abundances. When using mean site abundance, generalized linear models were implemented in R v. 4.0.4 (glm function, stats package), first with mean site abundance as the response variable, occupancy as a covariable and a Gaussian error structure, and second, with occupancy as the response variable, mean site abundance as covariable and a binomial error structure. When using individual site abundances, generalized linear mixed models (glmer function, lme4 package [52]) were implemented, first with individual site abundance as the response variable, occupancy as a covariate, island as a nesting factor of abundance and a Poisson error structure, and second, with occupancy as the response variable, individual site abundance as a covariate, island as a nesting factor of abundance and a binomial error structure. AORs were expressed as a logistic regression between the occupancy and the log of the abundance per species (mean site abundance per species). To examine the effect of both dispersal ability and niche breadth on AORs, a generalized linear model was constructed (glm function, stats R package), considering occupancy as the response variable and following a binomial error structure. As predictor variables, the log of the abundance (mean site abundance) and either dispersal ability or niche breadth, as well as their interaction, were considered. The best model was selected based on Akaike information criteria (AIC; aictab function, AICcmodavg package [53]). Additionally, four generalized linear models were constructed after subsetting the data by dispersal ability (ballooning and non-ballooning) and niche breadth (specialist and non-specialist) to calculate slope, intercept and McFadden *R*^2^ of the models for each species category. Models were performed across all islands, and within each island individually. A chi-squared test was used to test for potential association between dispersal ability and niche breadth (chisq.test function, stats R package).

### 2.4. Phylogenetic analyses of non-independence for abundance and occupancy

As both abundance and occupancy patterns may be influenced by shared common ancestry among species within a given assemblage [3], we test for phylogenetic non-independence using Blomberg's *K* statistic, a measure of the strength of phylogenetic signal of a given trait [54]. Values of *K* = 1 or greater imply that variation in the species trait being tested follows expectations under a Brownian motion model of evolution along a candidate tree, suggesting strong phylogenetic signal and thus phylogenetic dependence for the trait. Contrary to this, lower values for *K* imply phylogenetic independence of a given trait. A phylogeny was constructed using a backbone matrix comprising 4570 sequences for 1450 species retrieved from Macías-Hernández *et al*. [55], which includes (i) the concatenated gene matrix of Wheeler *et al*. [56], (ii) sequences bioinformatically extracted from the original transcriptome data in Fernández *et al*. [57], and (iii) COI and 28S sequences from species of the Iberian Peninsula, Madeira and Azores. Additionally, a local matrix comprising COI and 28S concatenated sequences for each species in this study was added to the backbone matrix. A region of the 28S rRNA gene was amplified using the primers 28S-O and 28S-B [58,59], using conditions described in Macías-Hernández *et al*. [55]. The phylogenetic tree was inferred using maximum-likelihood implemented in the program RAxML-HPC2, remotely run on XSEDE at the CIPRES Science Gateway (www.phylo.org), with the topology of Fernández *et al*. [57] used as a constraint. The concatenated matrix was partitioned by gene, and an unlinked GTR + CAT substitution model was assigned to each gene. The resulting phylogeny was made ultrametric (congruify.phylo function, geiger R package) [60], and the dated phylogeny of Fernández *et al*. [57] was used as a reference to provide fixed calibration points to date the target phylogeny using the program treePL [61]. The tree was then pruned (prune.sample function, picante R package) [62] to include only species present in the local community (123 species). Blomberg's *K* was then calculated for both species abundance and occupancy at archipelago scale, using the function ‘Kcalc’ in the ‘picante’ R package [62].

## 3. Results

### 3.1. Field sampling, DNA sequencing, dispersal and niche classification

A total of 21 082 spider individuals were collected and classified into 148 PUs (no type II PUs were recovered, i.e. all PUs could be cross referenced among plots based on taxonomy), from which 3338 individuals were selected for sequencing, yielding a total of 2663 sequences, representing a sequencing success rate of 79.7%. Implementing an intraspecific divergence threshold of 6.8% [36] yielded 119 lineages from which 123 species were inferred. One hundred and twenty of the 148 PUs correspond to a single PBS and the assignation of unsequenced individuals to PBS was unambiguous. For the remaining 18 PUs assigned to more than one PBS, unsequenced individuals per plot were assigned to a PBS following the proportion estimated for the corresponding plot using sequenced specimens. Four lineages yielded more than one species: (i) *Agyneta rurestris* (C. L. Koch, 1836) and *Agyneta fuscipalpa* (C. L. Koch, 1836); (ii) *Walckenaeria alba* Wunderlich, 1987 and *Walckenaeria afur* Thaler, 1984; (iii) *Pholcus gomerae* Wunderlich, 1980 and *Pholcus sveni* Wunderlich, 1987; (iv) *Walckenaeria gomerensis* Wunderlich, 1987 and *Walckenaeria cf. hierropalma* Wunderlich, 1987. Sixty species (49%) were classified as having limited dispersal ability (i.e. non-ballooner), with the remaining 56 having high dispersal ability (i.e. ballooner). Regarding niche breadth, the panel of experts (step 1) was able to classify a total of 77 species (63% of the total) as being either specialist or non-specialist, with 60 of them being consensually classified. For the 30 species classified as specialist species in step 1, the percentage of occupied cells corresponding with Q50 was 86.7%. Percentages of occupied cells for the other quantiles were Q10 = 62.8%; Q25 = 75%; Q75 = 100%. After the classification of species in step 2 using the 86.7% threshold (Q50), a total of 99 species (80%) were categorized; 63 species (51%) were classified as non-specialist, and 36 species (29%) as specialist. The remaining 24 species (19%) could not be categorized for niche breadth either because: (i) they were identified only to genus level, or (ii) they belong to potentially undescribed species whose distributions are unknown. Among the 63 non-specialist species, 37 (59%) were ballooners and 26 (41%) non-ballooners, while among the 36 specialist species, 20 (55%) were ballooners and 16 (45%) non-ballooners (see electronic supplementary material, table S2 for further details).

### 3.2. Richness, occupancy and abundance

**Figure 2.**
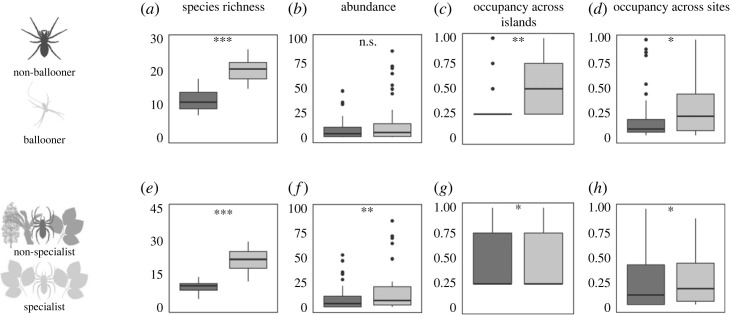
Box plots showing archipelago-scale differences for species richness (*a,e*), mean site abundance (mean number of individuals per site) (*b,f*), occupancy (proportion) across islands (*c,g*) and across sites (*d,h*), for non-ballooning (dark grey) and ballooning species (light grey) in the upper panels, and non-specialist (dark grey) and specialist species (light grey) in the lower panels. Chi-squared (d.f. = 1): ***p < 0.001, **p < 0.01, *p < 0.05, n.s. = not significant.

Regarding phylogenetic signals of abundance and occupancy, Blomberg's *K* values for both abundance (*K* = 0.274) and occupancy (*K* = 0.289) were less than one, suggesting that neither are phylogenetically constrained. A Chi-squared test did not provide evidence for association between dispersal ability and niche breadth (X-squared = 0.05, d.f. = 1, *p*-value = 0.83). Across all sites, species richness is significantly higher for ballooning species compared with non-ballooning species (*p* < 0.001, figure 2*a*). There was no significant difference (*p* = 0.09) for local species abundance using mean site abundance at the archipelago scale when comparing between ballooning and non-ballooning species (figure 2*b*, electronic supplementary material, table S3). Comparing between ballooning and non-ballooning species, abundances were also not significantly different at the scale of individual islands (*p* > 0.05 in all cases) (electronic supplementary material, figure S1a–d). In contrast, analyses using site-level abundance data (i.e. values of abundance per species, where *n* = the number of occupied sites) resulted in significantly higher abundance for ballooning species (*p* < 0.001), at both the archipelago and island scale (electronic supplementary material, figure S2a–e). Occupancy at both archipelago and island scales were recovered as significant predictors (*p* < 0.001) of abundance (electronic supplementary material, table S3) using both measures of abundance. There was, however, a significant occupancy difference between the two dispersal categories when accounting for mean abundance as a covariate, with ballooning species inhabiting more islands (*p* = 0.002) and more sites across the archipelago (*p* = 0.03), compared with non-ballooning species (figure 2*c,d*). At the scale of individual islands, there were no significant differences in site occupancy (number of sites) between ballooning and non-ballooning species for any of the islands (*p* > 0.05 in all cases) (electronic supplementary material, figure S3a–d). When accounting for site-level abundance as a covariate, there were no significant differences in the proportion of site occupancy between ballooning and non-ballooning species at the archipelago scale (*p* = 0.13) (electronic supplementary material, table S3). However, for all islands, there was a significant difference (*p* < 0.001), with ballooning species occurring at a higher number of sites (electronic supplementary material, figure S4a–e).

Regarding niche breadth, the results were similar across all measures of niche breadth categorization. Hereafter, we only refer to the Q50 threshold while the remaining results are found in the electronic supplementary material, tables S3 and S4. Across all sites, species richness is significantly higher for specialist species compared with non-specialist species (*p* < 0.001, figure 2*e*). Specialist species were significantly more locally abundant (using both mean site abundance and site count data) than non-specialist species across the archipelago (*p* < 0.001) (figure 2*f*; electronic supplementary material, figure S2f). At the scale of individual islands, specialist species were also significantly more abundant than non-specialist species (*p* < 0.05 in all cases) except for El Hierro using mean abundance data (electronic supplementary material, figures S1g–j and S2*g–j*). When accounting for mean abundance as a covariate, there were significant differences between specialist and non-specialist species with regard to the proportion of islands occupied (*p* = 0.03) (figure 2*g*), as well as the proportion of all sites at the archipelago scale (*p* < 0.001) (figure 2*h*). Within islands, there were no significant differences in site occupancy between specialist and non-specialist species (*p* > 0.05 in all cases) (electronic supplementary material, figure S3g–j). When accounting for site-level abundance as a covariate, there was a significant difference between specialist and non-specialist species at the archipelago scale (*p* < 0.001) as well as in the island of La Palma (*p* < 0.001) (electronic supplementary material, figure S4*f,h,i*), with abundance being higher in non-specialists. In Tenerife, La Gomera and El Hierro there were no significant differences (*p* > 0.05; electronic supplementary material, figure S4*g,j*). Both mean and site-level abundance were always recovered as significant predictors of occupancy for both the dispersal and niche comparison (*p* < 0.001; electronic supplementary material, table S3).

### 3.3. Abundance–occupancy relationship

**Figure 3.**
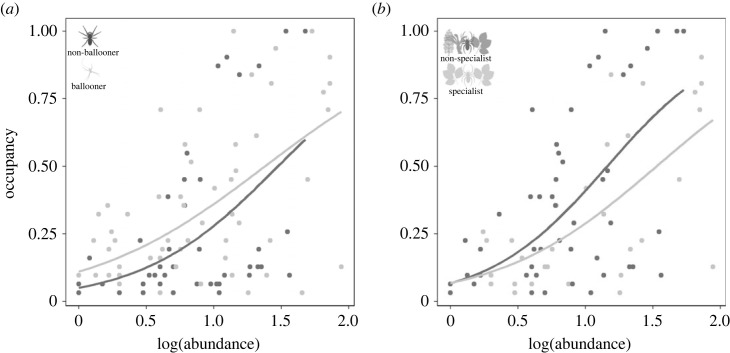
AORs for (*a*) ballooning and non-ballooning species and (*b*) specialist and non-specialist species, at the archipelago scale. Abundance of each species was measured as the mean local abundance among sites where it was sampled, while occupancy was considered the proportion of sites where the species is present. Abundance was log-transformed.

At the archipelago scale, generalized linear models revealed that the log transformation of abundance was a significant predictor of occupancy (*p* < 0.001), both when variation in dispersal ability and variation in niche breadth are accounted for (table 1). Individual models for species categories (ballooning, non-ballooning, specialist and non-specialist) revealed a positive relationship between species occupancy and local abundance at the archipelago scale in all cases (table 2). Dispersal ability was found to be a significant predictor of occupancy (*p* < 0.001). Niche breadth was recovered as a non-significant predictor of occupancy (*p* = 0.938). The linear model that accounted for both variables revealed both dispersal ability (*p* = 0.46) and niche breadth (*p* = 0.13) to be non-significant (table 1). AIC results revealed the most suitable model to be that accounting for both dispersal ability and niche breadth (ΔAICc = 0.00) (electronic supplementary material, table S4). AOR plots revealed that for a given value of local abundance, occupancy was greater for ballooning than for non-ballooning species as well as for non-specialist compared with specialist species (figure 3). There was evidence for a statistical difference between slopes for both niche breadth (*p* = 0.003) and dispersal ability (*p* = 0.005) as well as for niche breadth (*p* < 0.001) when both variables are considered in the model (table 2). Differences between dispersal categories seem to be attributed to differences in the intercept, which was higher for ballooning species (table 2). Generalized linear models at the island scale showed the same pattern as at the archipelago scale, with log-abundance being a strong predictor of occupancy (*p* < 0.001), with neither dispersal ability nor niche breadth being significant predictors (*p* > 0.05 in all cases), with the exception of El Hierro. In El Hierro, dispersal ability (*p* = 0.04) and niche breadth (*p* = 0.04) are significant predictors, but become non-significant when both variables are considered in the model (dispersal: *p* = 0.33; niche breadth: *p* = 0.32) (electronic supplementary material, table S5). Log-abundance and occupancy were positively related in all cases, with strong support (*p* < 0.001), with the exceptions being ballooning and specialist species in El Hierro (electronic supplementary material, table S6). There was no evidence for a statistical difference between slopes (*p*-values of the interaction > 0.05), except for El Hierro for both dispersal (*p* = 0.01) and niche breadth (*p* < 0.001) as well as niche breadth (*p* = 0.02) when both variables are considered in the model (electronic supplementary material, table S5). In Tenerife, for a given level of abundance, occupancy is higher for ballooning species but similar between specialist and non-specialist species. In La Gomera, ballooning species tend to occupy more sites at any given value of occupancy, while for niche breadth, at lower values of abundance, specialist species tend to occupy more sites. In La Palma and El Hierro, at lower values of abundance, ballooning species tend to occupy more sites, while at higher values of abundance, they tend to occupy fewer sites than non-ballooning species. In La Palma, for any given value of abundance, non-specialist species tend to occupy more sites than specialist species, whereas in El Hierro, for lower values of abundance, occupancy is higher for specialist species while at higher values of abundance, non-specialist species occupy more sites (electronic supplementary material, figure S5).

**Table 1.** Generalized linear models at the archipelago scale, examining the effects of (*a*) dispersal ability and (*b*) niche breadth on the AOR. Site occupancy (proportion of sites) was treated as the response variable. Log-transformed species abundance (mean site abundance) and either dispersal ability or niche breadth (category) were considered as predictor variables. Significant *p*-values (*p* < 0.05) are highlighted in italics. R syntaxis: glm(cbind(success, failures)∼log(abundance) × dispersal × niche, family = 'binomial’).

	(*a*) dispersal ability				(*b*) niche breadth				(*c*) dispersal ability × niche breadth			
	estimate	s.e.	*z* value	*p*(>|*z*|)	estimate	s.e.	*z* value	*p*(>|*z*|)	estimate	s.e.	*z* value	*p*(>|*z*|)
intercept	−2.082	0.099	−20.960	*<0.001*	−2.625	0.117	−22.425	*<0.001*	−2.625	0.157	−16.762	*<0.001*
log-abundance	1.510	0.092	16.381	*<0.001*	2.262	0.121	18.745	*<0.001*	2.549	0.175	14.542	*<0.001*
dispersal ability	−0.849	0.176	−4.840	*<0.001*					−0.183	0.246	−0.743	0.457
niche breadth					0.016	0.211	0.078	0.938	0.415	0.277	1.498	0.134
log-abundance : dispersal	0.477	0.169	2.824	*0.005*					−0.401	0.248	−1.613	0.107
log-abundance : niche breadth					−0.552	0.185	−2.984	*0.003*	−1.064	0.242	−4.395	*<0.001*

**Table 2.** Intercept, slope and *R*^2^ of linear models at the archipelago scale for occupancy (response) versus log-abundance (predictor) for different subsets of species based on dispersal ability and niche breadth. Significant *p*-values (*p* < 0.05) are highlighted in italics.

	intercept	*p*-value Intercept	slope	*p*-value slope	McFadden *R*^2^
ballooning	−2.0816	*<0.001*	1.5101	*<0.001*	0.3052
non-ballooning	−2.9312­	*<0.001*	1.9871	*<0.001*	0.2846
specialist	−2.6085	*<0.001*	1.7093	*<0.001*	0.3647
non-specialist	−2.6249	*<0.001*	2.2618	*<0.001*	0.3861

## 4. Discussion

### 4.1. Influence of dispersal and niche breadth on species abundance and occupancy

Dispersal facilitates the colonization of new patches of habitat for resource exploitation [63], thus increasing patch occupancy and enhancing connectivity among populations. Consistent with this, we find statistically significant differences in occupancy between ballooning and non-ballooning species across all sites (figure 2*c,d*). However, in contrast, there were no significant differences in abundance between the two dispersal categories for mean site abundance (figure 2*b*). At the scale of individual islands, there were no significant differences for abundance or occupancy between the two dispersal categories (electronic supplementary material, figures S1 and S3). Although significant differences were found when considering abundance and occupancy measures at the level of individual sites at both scales (electronic supplementary material, figures S2 and S4), it seems likely that these results are influenced by a taxonomic bias. The 20 highest site-level abundances involved only six species, all of them ballooning species. Mean abundance across sites for each species is a less biased estimator, and conclusions are therefore based on this measure. These results highlight how variation in dispersal ability influences species occupancy within a spatial scale and landscape-dependent context. At smaller spatial scales (within islands), within habitat landscapes that are likely to have been largely continuous through time [26,64], dispersal limitation among spider species would appear to be insufficient to limit occupancy, relative to more dispersive species. However, at a larger spatial scale (among islands), within which habitat patches are separated by large areas of uninhabitable landscape (ocean), the influence of dispersal limitation becomes apparent. At this spatial scale, dispersal limitation acts to limit movement among patches, and a clear signature of higher site occupancy is associated with species with higher dispersal ability (ballooning species). Overall, ballooning categorization serves as a robust proxy for passive dispersal, in line with previous work (e.g. [42–44]), with ballooning enhancing patch occupancy across the metacommunity. Regarding abundance, previous work (e.g. [6–8]) have found that species with higher dispersal ability were significantly more abundant compared with species with dispersal limitation, at both broader and narrower geographical scales. We found no significant differences in mean abundance comparing ballooning and non-ballooning spiders species, consistent with Levine & Murell [14], who have suggested that differences in dispersal ability are unlikely to influence abundance patterns at metapopulation scales. As dispersal ability influences the rate of individual arrival, its influence in shaping abundance patterns is likely to be more determinant in the early stages of habitat colonization, such as after disturbance, or the emergence of new habitat [65,66]. Given the origin of the youngest island in the study system, El Hierro, is relatively old (estimated to date back 1.12 million years, [67]), and the relative habitat stability of the laurel forest through time ([58,59] but see [31]), both factors may underpin similar local abundance patterns between dispersal-limited and dispersive species. Further studies are needed in order to understand the temporal scale and disturbance dynamics at which spider dispersal ability could constrain species local abundance.

Niche differences among species can play a key role in structuring communities through processes such as competitive displacement and habitat partitioning [68]. In contrast with dispersal ability, comparisons between habitat specialist and non-specialist species revealed statistically significant differences for abundance, but not occupancy. Compared with specialist species, non-specialist species were found to occupy a similar number of habitat patches (islands) and sites across the entire archipelago (figure 2*g,h*), and within islands they occupied a similar number of sites (electronic supplementary material, figure S3). However, non-specialist species displayed lower abundances, compared with specialist species, at both archipelago and island scales (figure 2*f*). These results provide support for the ‘niche position’ [18] and ‘jack-of-all-trades is a master of none’ hypothesis [22], which implies that specialist species perform better than non-specialist (generalist) species in their optimal habitats, at the expense of a poor performance in other suboptimal habitats [69]. An interesting feature of our data is that, in contrast with Verberk *et al*. [68], who found specialist species to be less dispersive relative to non-specialist species, we find similar proportions of ballooning species between the two niche breadth categories (56% in non-specialists and 60% in specialists), thus contrasting with the suggestion that habitat specialists are likely to be more sedentary, due to adaptation to stable habitats [68].

### 4.2. Influence of dispersal and niche breadth on the abundance–occupancy relationship

Results of the linear models support the general ecological pattern of the AOR [2–4], as log-abundance and occupancy were always significantly positively correlated, independently of geographical scale (table 1; electronic supplementary material, S1 and S2). Dispersal ability was found to be a strong predictor of the AOR, whereby across all sites for a given level of local abundance, occupancy is higher for ballooning species than for non-ballooning species (figure 3*a*), as predicted by Gaston & Blackburn [9], and consistent with higher dispersal ability contributing to higher occupancy (figure 2*c,d*). Slopes were statistically different among dispersive categories, being higher for non-ballooning species. The *y*-intercept was found to be higher for ballooning species. Contrasting with our findings, both Foggo *et al*. [16] and Sahara *et al*. [17] found no significant differences in AOR slopes when comparing dispersal-limited and dispersive species in the marine realm. However, they did recover a higher *y*-intercept for dispersive species providing more explanation for differences in linear trends. Using four different phyla of marine invertebrate taxa, Foggo *et al*. [16] revealed a higher fit of the AOR for taxa with planktonic dispersal (good dispersers) compared with taxa with non-planktonic dispersal (poor dispersers). They also found that, for a given level of abundance, occupancy was higher for planktonic dispersal. In their analyses of AORs across species of rocky intertidal gastropod, Sahara *et al*. [17] also found that regional occupancy was higher for a given level of abundance in planktonic species, resulting in a less aggregated distributions compared with non-planktonic species.

Foggo *et al*. [16] propose differences in fecundity, mediated by body size, as a potential explanation for a higher fit to the AOR for more dispersive species. They hypothesize that planktonic species (good dispersers) are prone to produce more offspring than non-planktonic (poor dispersers) due to their larger body size. Higher offspring production increases the probability of population establishment through increased opportunity to encounter suitable environmental conditions [70]. Thus, due to higher propagule pressure, good dispersers are more widespread at any given level of abundance. Sahara *et al*. [17] additionally propose that a dampening effect of larval dispersal can reduce local extinction risk, thus increasing patch occupancy and decreasing spatial patchiness at the metapopulation scale. While it is widely acknowledged that different life stages of smaller spider species and the early juvenile instars of larger species disperse by ballooning [48,71], the relationship between body size, offspring density and ballooning remains unknown in the case of spiders. If larger species produce more offspring and disperse by ballooning, they may be expected to be more abundant in ballooning studies. However, families with larger species are usually absent or at extremely low frequencies (less than 1%) in ballooning studies, with smaller species being more abundant (e.g. [42,65]). This suggests that ballooning propensity is not correlated to differences in fecundity driven by body size, as suggested for marine invertebrates by Foggo *et al*. [16]. In the case of spiders, ballooning appears to be phylogenetically conserved across spider families [72,73]. Although it is unknown why some families exhibit higher ballooning frequencies than others, it has been suggested that it may be related to microhabitat requirements, with non-ballooning lineages being typically associated with more specialized narrow niches (e.g. species strongly linked to edaphic or underground environments) that are less susceptible to convective air currents [72]. Our results are suggestive of a model for spiders where propagule pressure is higher in ballooning species, potentially driven by a higher exposition to passive dispersal vectors, rather than by body size or offspring density differences, leading to higher occupancy for a given level of abundance for ballooning species. However, more understanding is needed with regard to the suggested relationship between dispersal and other species traits.

Niche breadth was found to be a significant predictor of the AOR, with specialist species occupying more sites at any given value of abundance (figure 3*b*). Our results fit with Verberck *et al*. [68], who found that for a given value of occupancy, specialists were more abundant than non-specialists, with the AOR for non-specialist species also providing a higher fit to the data than that for specialist species. Verberck *et al*. [68] suggest that this difference may be explained by specialist species being more affected by the spatial distribution of environmental conditions. Comparing our results with Verberck *et al*. [68] is complicated, because they implemented abundance as a dependent variable and occupancy as a covariate, whereas in our analyses, occupancy was considered as the dependent variable. There are also contrasting approaches between Verberck *et al*. [68] and this study, with regard to the classification of specialist and non-specialist species. Verberck *et al*. [68] were able to derive estimations of niche breadth for species, thus allowing the comparison of patterns between species that are specialized within a habitat, to those that are not. Our classification is more coarse-grained, where species are considered specialist if they are largely limited to the habitat, and non-specialist if they occur in other habitats. Thus, it remains plausible that variation in niche specificity among species within the laurel forest habitat may explain variation in AORs. Our research has focused on a single habitat type and, while logistically challenging, expanding the scope of sampling across multiple habitat types would enable broader estimations of niche specialization [51,74], including not only niche breadth but also species niche position.

## 5. Conclusion

Our results highlight the relevance of both dispersal ability and niche breadth for understanding patterns of abundance and occupancy among spider species, together with the landscape-scale context of habitat suitability within which both variables act. Higher dispersal ability favours site occupancy within landscapes of discontinuous habitat (archipelago level) but appears to be less consequential within continuous habitat (within islands). Specialist species appear to be more abundant than non-specialists, supporting the ‘jack-of-all-trades is a master of none’ hypothesis. We found a strong relationship between log-abundance and occupancy, with significant effects of both niche breadth and dispersal ability on the AOR, where good dispersers and non-specialists occupy more sites for a given level of abundance. Our community-scale framework highlights the importance of spatial scale when seeking to understand what factors shape species abundances and occupancy, with particular reference to spatial patterns of landscape variation. Our results thus suggest that taking into account niche landscapes of individual species will lead to improved understand of the AOR. Further studies integrating dispersal ability, niche and habitat landscape are needed for a fuller understanding of AORs and the variation they present.

## Data Availability

The information associated with the studied species, including values of abundance and occupancy as well as DNA sequences used for estimating phylogenetic independence, are available from the Dryad Digital Repository: https://doi.org/10.5061/dryad.4f4qrfjhb [75]. All supplementary tables and figures cited in the main text have been uploaded as electronic supplementary material [76].
